# An Asymmetric Investigation of Remittance and Trade Openness Impact on Inequality: Evidence From Selected South Asian Countries

**DOI:** 10.3389/fpsyg.2021.720887

**Published:** 2021-10-08

**Authors:** Liu Fang, Md. Qamruzzaman

**Affiliations:** ^1^College of Economic and Management, Qinghai Minzu University, Xining, China; ^2^School of Business and Economics, United International University, Dhaka, Bangladesh

**Keywords:** inequality, trade openness, remittance, NARDL, asymmetry causality JEL classification Code: 015, F24, P33, I14

## Abstract

This study’s motivation is to explore the relationship pattern between remittance, trade openness, and inequality of selected south Asian countries for the 1976–2018 period. The study performed non-linear tests, including unit root tests, non-linearity applying ordinary least squares (OLS) and BDS tests, non-linear autoregressive distributed lagged (NARDL) tests, and asymmetry causality tests to assess their association. Study findings with non-linear unit root tests suggest that the research variables follow the non-linear process of becoming stationary from non-stationary. The non-linear OLS and BDS test results confirm the existence of non-linearity among research variables, implying rejection of the null hypothesis of “no non-linearity.” Furthermore, the results of the Wald test in NARDL confirm the availability of asymmetric links among variables. Besides this, the results of NARDL confirm the long-run asymmetric relationship between remittances, trade openness, and inequality in all sample nations. Findings suggest that both positive and negative shocks in remittances and trade openness is critical to either instituting or vexing the present state of inequality in the economy in the long term. In the directional relationship with asymmetry causality, the study shows that the feedback hypothesis holds to explain the asymmetric causal effects that are positive shocks in remittances and trade openness toward inequality.

## Introduction

Throughout the developing world, policymakers are interested in devising new strategies for rebalancing skewed income distributions and reducing poverty. The choice of such strategies crucially hinges on an improved understanding of the sources of income inequality (Shams and Kadow, 2020). Why do certain types of incomes go to particular groups of people? Moreover, what roles do variables, such as land ownership, migration, and education, play in improving income distribution and lifting people out of poverty? Furthermore, another major concern of social sciences for more than a century has been how injustice is created and reproduces over time. However, the connection between injustice and the mechanism of economic growth is far from well understood (Aghion et al., 1999; Islam and McGillivray, 2020). The impact of income and wealth disparity on socioeconomic influences has been the primary interest of social science (Kim et al., 2020; Bergstrom, 2020; Seo et al., 2020). The empirical literature is identified to support that income allocation plays a significant role in economic development. The role of income and wealth disparity has long been a significant concern of social sciences. The research on the relationship between income distribution and economic growth can at least be traced back to Kaldor (1956), who postulated the impact of income distribution on capital accumulation and, hence, economic growth. In the same period, the development of economic literature continues the seminal work of Kuznets (1955), which focuses mainly on the opposite direction, i.e., the impact of growth or the stage of development on income distribution.

Inequality is a state of the economic situation resulting from a difference in the individual endowment. In the recent period, inequality regains researchers, academicians, and policymakers’ attention due to any given level of natural or human capital; the more inequitable its distribution, the higher the poverty one could expect (Balisacan and Ducanes, 2006). Furthermore, according to Stiglitz (2012), inequality negatively affects society by increasing social costs through poor education, healthcare, and occupation. Again, social imbalance causes corruption, nepotism, criminality, and many others. Therefore, the state of inequality is subject to crucial concern due to its versatile effect on the economy; in this connection, empirical literature provides evidence that the researchers and policymakers wish to disclose the critical macrofundamentals that can play a crucial role in mitigating the gap in the economy (Seo et al., 2020).

Non-classical growth theory advocates that efficient capital mobility might play a deterministic role in reducing inequality. Trade internationalization is one of the paths. In Suci et al. (2016) and Nguyen (2020), they establish that trade liberalization negatively affects inequality, implying that reducing the income gap in the economy creates opportunities in income accumulation, redistribution of income, and employment. Similar effects are also available in Borraz and Lopez-Cordova (2007), Almas and Sangchoon (2010), Faustino and Vali (2011), Gourdon (2011), Salimi et al. (2014), Amjad (2015), and Bukhari and Munir (2016) claims that trade liberalization increases inequality in highly educated, great countries, whereas there are diminishing effects also in primary educated generous countries. However, it increases inequality in non-educated generous countries, suggesting that this part of the population does not benefit from trade openness because it is not included in export-oriented sectors. It is ubiquitous that people move from their home country to others with a perception of increasing living standards by grabbing higher purchasing power (Koechlin and Leon, 2007). The relationship between migrants and remittance is that migrant families receive money as an alternative source of income, and this induces them to increase their living standards. Among all macrofundamentals, the role of foreign remittance in income inequality importantly appears in the empirical literature (Dreher et al., 2010). Remittances constitute an essential external financing source for many emerging markets and developing economies at the macrolevel. At the microlevel, they can facilitate investments in health, education, or small businesses. An extensive literature documents their beneficial effects on poverty and inequality yet to unleash with convincingly.

**FIGURE 1:**
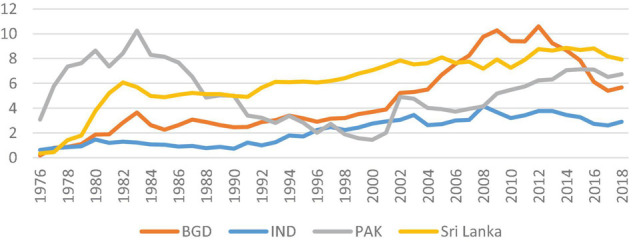
Remittance inflows as a percentage of GDP from 1976 to 2018. Source: author calculation by using WDI data set.

In the year 2018, the ratio of remittance inflows to the GDP of South Asian countries was exhibited as Bangladesh (5.67%), India (2.89%), Pakistan (6.73%), and Sri Lanka (7/92). Considering the pattern of remittance inflows in South Asian countries, it is evident that a declining nature is observable from 2010 to 2017 (see Figure 1). However, the year 2018 shows growth in remittance inflows in the economy. This is because foreign remittance, mostly migrant worker remittance inflows, is a pivotal ingredient in the capital accumulation process by supplying much-needed money flows in the economy (Edwards and Ureta, 2003; Acosta et al., 2006; Zhunio et al., 2012).

This study is novel in different aspects. First, South Asia is an exciting focus for studying inequality, not just because it accounts for the bulk of the world’s population, but also because of its constituent countries’ various experiences concerning inequality and growth. For South Asia, the studies reviewed in this paper show all countries as having had recent experiences of rising inequality (India in the 1990s, Pakistan in the late 1980s, Bangladesh in the first half of the 1990s, Nepal from the mid-1980s to the mid-1990s, and Sri Lanka over the past three decades). Furthermore, South Asia’s migration significantly affects remittances because millions of highly and semiskilled people work in Western and Gulf nations. Remittances are a significant element in South Asian economics because they provide subsistence for impoverished people through a beneficial effect on capital creation. We may conclude from the available data that remittances aid Asian nations through natural disasters, such as the tsunami in Sri Lanka, the earthquake in Nepal, and the global economic crisis of 2007/8. The selection of these four countries is based on various criteria, including family income in the origin country, economic conditions, migratory destination, immigrant economic status, political situation, and geographic region.

Second, the stationary process is investigated with a non-linear unit root test following Kapetanios et al. (2003) and Kruse (2011); furthermore, non-linearity is tested by applying the non-linear OLS and BDS tests. Third, long-run asymmetry is investigated by following the non-linear framework proposed by Shin et al. (2014) and directional causality established with an asymmetry causality test following the proposed framework by Hatemi-j (2012).

Study findings suggest that remittance inflows, trade openness, and the measure of inequality exhibit stationarity by following non-linear processes. Besides this, non-linearity also confirms by the estimation of non-linear ordinary least squares (OLS) and BDM tests. Furthermore, considering the results of the non-linear autoregressive distributed lagged (NARDL) test, the standard Wald test results establish long-run asymmetry between remittance inflows, trade openness, and inequality. Finally, the directional causality output follows the asymmetry causality test proposed by Hatemi-j (2012).

The remaining structure of the paper is as follows. Section II exhibits a summary of the relevant literature on the current study. A detailed explanation of research variables and econometric methodologies is inserted in Section III. Section IV deals with empirical model estimation and interpretation. Finally, the study ends with a summary of findings in Section V.

## Literature Review

### Nexus Between Inequality and Remittance Inflows

Remittances are the money and goods transferred to families back home by migrant workers employed outside of their origin communities. Although about 250 million people, or 3.4% of the world population, live in countries where they were not born (World Bank, 2019), migration and remittances have attracted increasing attention globally over the past decades. Remittances are considered as more stable external income for developing countries rather than other private flows and foreign direct investment (FDI) and have been observed to be increased significantly during the time of economic depression and financial crisis (Bui et al., 2015). The extant literature on the economic effects of remittances is inconclusive. Many studies find that remittances have a positive impact on economic growth and development (Catrinescu et al., 2009; Feeny et al., 2014; Hatemi-J and Uddin, 2014), stimulate financial developments (Chowdhury, 2011; Qamruzzaman and Jianguo, 2020b), and increase investments (Zhu and Luo, 2010; Lartey, 2013). Although some studies show that remittances reduce income inequality (Qamruzzaman et al., 2019), others find that such transfers deteriorate (Acosta et al., 2006) or have no effect on inequality (Brown et al., 2013; Beyene, 2014). According to Stark et al. (1986) and Durst and Ståhle (2013) remittances increase income inequality because it is the wealthy households that assist their family members to migrate most compared with poorer households.

The nexus between foreign remittance and inequality is one of the causal relationships immensely attracting researchers, academicians, and development agencies since the 1980s; see, for instance, Stark et al. (1986) and Adams (1991). A study conducted by Ahmed et al. (2020) assesses the impact of remittance on income inequality in Bangladesh considering household income and expenditure survey data. The study applies quantile regression for exposing the causal effects running from remittance to income inequality. Study findings reveal that that remittance from both domestic and international migrants improves expenditures. However, they have different impacts on income inequality. Although internal remittances are more likely to reduce household income inequality, international remittances increase it significantly.

It is apparent in the empirical literature that a growing number of empirical studies are conducted in this regard. Taking account of empirical evidence, we observe three lines of findings available. First, the positive effect of foreign remittance inflows on inequality studies finds that migration and remittances increase inequality (Adams, 1991, 2006; Barham and Boucher, 1998; Rodriguez, 1998; Adams et al., 2008a; Lokshin et al., 2010; Möllers and Meyer, 2014; Bouoiyour and Miftah, 2015; Bouoiyour and Miftah, 2018; Kousar et al., 2019; Chea, 2021; Tokhirov et al., 2021). They argue that remittance inflows in the economy increase recipient groups’ purchasing power, implying that having excess money for consumption in the situation remittance recipient’s relative changes social position compared with the poor and tried to match their consumption with a rich group. It is hypothesized that a household’s perception of its income through remittance is a major component because it determines the impact of remittances on welfare: a significant role of remittances in replacing contributions made by migrant workers and the necessity of them containing extra production information to make a significant impact on the welfare of the families. In a study, Bajra (2021) advocates that remittances and income inequality are closely linked although the effects of remittances on inequality are difficult to separate. Moreover, using a direct consumer remittance goal reduces the likelihood that the multiplier impact of remittances may be seen in all sectors of the economy.

Second, foreign remittance helps reduce inequality in the economy (Acosta et al., 2006; Brown and Jimenez, 2007; Pfau and Giang, 2009; Gubert et al., 2010; Zhu and Luo, 2010; Anwar and Mughal, 2012; Olowa and Shittu, 2012; Margolis et al., 2013). Third is the neutral effect running from remittance inflows to inequality (Yang and Martinez, 2006; Yang, 2011; Beyene, 2014).

Apart from using macrolevel data, a group of researchers investigates the impacts of remittance on inequality using household-level data. For example, Howell (2017) performed a study dealing with migrants’ remittance effects on ethnic group income inequality in China. Study results suggest that migrants’ remittance increases income inequality despite reducing spatial disparities. This finding implies that remittance recipients of the ethnic groups enjoy disproportional benefits compared with general people. A similar conclusion is also available in the study of Barham and Boucher (1998), Adams et al. (2008a), and Acharya and Leon-Gonzalez (2012) used household survey data in Nepal by applying the household consumption function. Study findings established that overall remittance inflows in the economy augment the prevailing situation of inequality.

*H1: Inflow of remittances in the economy positively assists in reducing inequality.*

### Nexus Between Inequality and Trade Openness

During the mid-1980s, trade liberalization emerged as a catalyst for globalization through technological expertise sharing and transferring across the cross-border country. During the globalization process, the continual flow of goods, services, and capital expedite economic growth by ensuring efficiency and optimal mobilization in the economy (Otmani and Abadli, 2019). As a result, the developing economy experiences many employment-generation opportunities, financial intermediation, and higher earning possibility. Therefore, in the empirical literature, the role of trade openness considering the macroeconomic phenomenon extensively investigated among those impacts on inequality is high. In the study of McCulloch et al. (2001), Erum et al. (2016), and Bong and Premaratne (2019), they postulate that trade openness effects could be observed in poverty. Still, the biggest one appears in inequality, which is derived from economic growth. The importance of inequality is explained by Kaldor (1957). He argues that economic growth fostered by additional investment in the rich people’s economy saves more and assists in capital accumulation in the long run.

Trade openness accelerates the speed of income inequality negative associations (see Milanovic, 2005; Bucciferro, 2010; Castilho et al., 2012; Bayar and Sezgin, 2017; Dilara and Çiğdem, 2021; Xu et al., 2021). The effect of trade openness on inequality is adverse due to several inherent economic attributes, such as well-endowed capital. Trade liberalization, according to Krugman and Elizondo (1996), decreases income disparities across the nations through economies of scale owing to market size. Furthermore, they explain that the total revenue of a place is a result of these centripetal and centrifugal forces that influence industrial location throughout a national area. Because there is a connection between these factors and trade liberalization, trade openness partly influences industrial location. Fujita et al. (1999) further establishes that trade integration might eventually reduce regional inequalities by drawing manufacturing to a country’s less developed regions, particularly when wages are lower in these remote places due to the country’s relative lack of labor mobility.

Another line of empirical studies available in explaining the positive association is that trade openness assists in reducing income inequality in the economy (Dağdemir, 2008; Vollrath, 2009; Székely and Sámano, 2012; Khan and Bashir, 2013; Wahiba, 2015; Andersson and Palacio Chaverra, 2016; Yenipazarli and Kucukkaya, 2016; Andersson and Palacio, 2017; Topuz and Dağdemir, 2020; Topuz and Dağdemir, 2020; Xu et al., 2021).

Furthermore, a group of researchers concludes with a neutral effect that is there no inclusive evidence running between trade openness and inequality (Edwards, 1997; Li et al., 1998; Higgins et al., 1999; Dollar and Kraay, 2002; Trabelsi and Liouane, 2013; Agusalim and Pohan, 2018).

In a study, Jalil (2012) suggests that when trade openness reaches a certain critical threshold, inequality increases with trade openness; however, when this critical threshold is passed, income inequality decreases even as trade openness increases. Furthermore, Calderón and Chong (2001) postulate that trade openness increases income inequality in necessary goods exports and reduces industrial goods exports.

*H1: Domestic trade expansion allows a higher standard of living, thus positively assisting in reducing inequality.*

### The Motivation of the Study

Considering the empirical literature findings, the nexus between remittance–income inequality and trade openness–income inequality is extensively investigated. However, non-linearity is ignored to our best knowledge; the study’s motivation is to mitigate the existing research gap by performing a non-linear investigation with several non-linear tools and techniques in the empirical literature. Moreover, study findings with the non-linear analysis create a new avenue for policymakers and researchers.

## Data and Econometric Methodology

### Data and Descriptive Statistics

Annual time series data over the period 1976–2018 utilizes empirical investigation and was collected from world development indicators of the World Bank (WB), Federal Reserve Bank of St. Louis (FRED), and International Financial Statistics of International Monetary Fund (IMF). As a dependent variable in the empirical estimation, inequality is measured by versatile proxy, including the GINI coefficient (Mekenbayeva and Karakus, 2011; Abba and Baba, 2014; Ali, 2014; Cheng, 2015; Ceesay et al., 2019), life expectancy (Tabassum and Majeed, 2008; Kamila and Baris, 2011; Ceesay et al., 2019). In the study, we consider the Gini coefficient a proxy of inequality extracted from Unu-Wider (2020). Other than the dependent variable, we have two independent variables: trade openness (TO) and remittance inflows (R). All the variables were transformed into a natural logarithm before estimation. Descriptive statistics of research units are displayed in Table 1.

**TABLE 1:** Descriptive statistics of research variables.

	**Mean**	**Median**	**Maximum**	**Minimum**	**Std. Dev.**	**Skewness**	**Kurtosis**	**Jarque-Bera**
**Panel A: for Bangladesh**								
IE	4.124507	4.145899	4.277388	3.899991	0.11508	–0.31601	1.762747	3.37794
R	1.278266	1.196617	2.359716	–1.68502	0.80057	–1.24227	5.875244	25.27
TO	3.292066	3.280556	3.873509	2.814678	0.332116	0.255234	1.7628	3.134672
**Panel A: for India**								
IE	4.104044	4.110998	4.236495	3.944103	0.087551	–0.14678	1.801089	2.666247
R	0.598869	0.806641	1.427583	–0.46944	0.57873	–0.25412	1.564076	4.060342
TO	3.187533	3.124261	4.021661	2.503014	0.529657	0.230473	1.541842	4.092719
**Panel A: for Pakistan**								
IE	4.119902	4.125615	4.203901	4.014959	0.055537	–0.24139	1.922922	2.438053
R	1.537044	1.613047	2.327047	0.37407	0.497715	–0.63913	2.568312	3.185496
TO	3.497187	3.508307	3.661238	3.231051	0.104963	–0.73333	3.041054	3.767328
**Panel A: for Sri Lanka**								
IE	4.268391	4.243188	4.339224	4.196585	0.044915	0.302531	1.552233	4.308725
R	1.676905	1.819225	2.182307	–1.01942	0.691344	–2.77758	10.43395	150.7157
TO	4.201726	4.225738	4.484543	3.836521	0.181984	–0.52351	2.097176	3.344812

### Methodology

In the study, we perform several econometric techniques of unveiling certain types of information. First, investigating variables in the order of integration, we applied both traditional unit root tests, namely, ADF (Dickey and Fuller, 1979), P-P (Phillips and Perron, 1988), and KPSS (Kwiatkowski et al., 1992), assuming a linear stationary process. Then, following Galadima and Aminu (2020) and Qamruzzaman and Karim (2020), we performed non-linear unit root tests proposed by Kapetanios et al. (2003) and Kruse (2011). Furthermore, non-linearity also checks by following (Broock et al., 1996) and the non-linear ordinary least squares (NOLS). Furthermore, the coefficient of non-linear effects positive and negative shocks of remittance and trade openness also gauge applying NARDL proposed by Shin et al. (2014). Furthermore, finally, asymmetric causal relationships are also investigated following the asymmetry causality tests propose by Hatemi-j (2012).

#### The Kapetanios Unit Root Test

There is a growing dissatisfaction with the standard linear ARMA framework, which investigators use to test unit roots (Kapetanios et al., 2003). Much of this arises because a theoretical prediction of stationarity in several economic areas is confounded in practice by the standard Dickey-Fuller (DF) test (Rose, 1988; Taylor et al., 2001). To resolve this issue related to the linear unit root test, Kapetanios et al. (2003) introduced an alternative of a non-linear exponential smooth transition autoregressive (ESTAR) process global stationarity.

Therefore, following Kapetanios et al. (2003), Liu and He (2010), Anoruo and Murthy (2014), and Galadima and Aminu (2020), the paper specifies the ESTAR model as


(1) $$△Y_{t}=\beta Y_{t-1}\left\{1-exp\left(-\theta Y_{t-1}^{2}\right)\right\}+\varepsilon_{t}\;t=1,2\ldots T,$$

where *Y_t_* is the demeaned or detrended time series of interest, β and *θ* are unknown parameters, the term $\left\{1-exp\left(-\theta Y_{t-1}^{2}\right)\right\}$ is the exponential transition function adopted in the test to represent the non-linear adjustment, and ε_*t*_, is the stochastic term assumed to be generally distributed with a zero mean and a constant variance.

Hence, from Equation (1), we test the following hypothesis:


(2) $$H_{0}:\theta =0$$

and


(3) $$H_{1}:\theta >0.$$

Obviously, according to Davies (1987), testing the null Hypothesis (1) directly is not feasible because β is not identified under the null. Resolving this issue, Kapetanios et al. (2003) suggests applying Luukkonen et al. (1988) and deriving the at-type test statistic. In addition to the reparameterization of Equation (1), obtain a first-order Taylor series approximation to the ESTAR model under the null and get the auxiliary regression.


(4) $$△Y_{t}=\delta Y_{t-1}^{3}+error,$$

suggesting that it is easy to get the value of *t-*statistics for *δ* = 0, *against δ* < 1 as


(5) $$t_{NL}=\frac{\hat{\delta }}{s.e.\left(\hat{\delta }\right)},$$

where $\hat{\delta }$ is the OLS estimate of d, and $s.e.\left(\hat{\delta }\right)$ is the standard error of the ^ d. Non-etheless, it is noteworthy that the *t*_*NL*_ statistic does not follow an asymptotic standard normal distribution.

#### The Kruse Nonlinear Test

Kapetanios et al. (2003) proposes the ESTAR-based non-linear unit root test to assume that the location parameter c in the smooth transition function is equal to zero (see Equation 1) for empirical study and became popular among researchers. However, a growing number of studies observes that the coefficient of *c* is significant, for example, Michael et al. (1997), Sarantis (1999), Taylor et al. (2001), and Rapach and Wohar (2006). In a study, Kruse (2011) argues that excluding basic assumptions leads to the non-standard testing problem. Therefore, modified test statistics are used to mitigate location parameter issues by following Abadir and Distaso (2007). Eventually, the following revised ESTAR specification was proposed:


(6) $$△Y_{t}=\alpha Y_{t-1}+\delta Y_{t-1}\{1-exp\left(-\theta {\left(Y_{t-1}-c\right)}^{2}\right\}+\varepsilon_{t}\;t=1,2\ldots T,$$

where *ε*_*t*_∼ iid (0, σ^2^). If the smoothness parameter γ approaches zero, the ESTAR model becomes a linear AR (1) model, i.e., *Y_t_* = *αY*_*t*−1_ + *ε*_*t*_ that is stationary if −2 < α < 0 non-linear OLS. Hence, the modified ADF regress is


(7) $$△Y_{t}=\sum_{j=1}^{p}\alpha_{j}Y_{t-j}+\gamma_{1}Y_{t-1}^{3}+\gamma_{2}Y_{t-1}^{2}+\varepsilon_{t}\;t=1,2\ldots T.$$

In the equation, the null hypothesis *H*_0_ : *θ* = 0 turns out *γ*_1_ = *γ*_2_ = 0 with the alternative hypothesis of *γ*_1_ < 0; *γ*_2_ ≠ 0, where *γ*_2_ stems from the fact that the location parameter “c” is allowed to take non-zero values. Therefore, according to Yıldırım (2017), a standard wild test is not appropriate for deriving test statistics; instead Kruse (2011) proposes a modified Wald test by integrating the procedure initiated by Abadir and Distaso (2007), which is widely known as “the Kruse” test in literature. That is,


(8) τ=tβ2=02+1(β^<0)tβ1=0 2

#### The Hatemi-J Asymmetry Causality Test

The causality test, according to Hiemstra and Jones (1994), to apply a linear assumption, possesses certain drawbacks: the incapacity of addressing non-linear effects from independent variables to the dependent variable. Therefore, following the Granger and Yoon (2002) empirical study, the cointegration test was executed using the decomposition of positive and negative shocks for the first time. Furthermore, taking an initial non-linear framework, Hatemi-j (2012) extends their work for investigating causality tests, hereafter known as asymmetry causality testing in the empirical literature. The proposed framework is referred to as asymmetry in the sense that the proposed framework is capable of detecting both positive and negative shock effects.

Following the pattern, study decomposes remittance inflows and trade openness into positive and negative shocks and puts considerable effort into seeing results that are a positive and negative variation of remittance inflows and trade openness on income inequality. It is presumed that positive and negative effects might have different impacts on income inequality (Hatemi-j, 2012).

To testify to the causality between positive and negative shocks in remittance inflows and trade openness on selected South Asian countries’ income inequality, the impact of the cumulative sum of effects can be expressed as follows:


(9) $$\left\lfloor \begin{array}{cc} IE_{t} & \\ R_{t}^{+} & \\ TO_{t}^{+} & \end{array}\right\rfloor =\left\lfloor \begin{array}{c} \alpha_{10}\\ \beta_{20}\\ \gamma_{30} \end{array}\right\rfloor +\left\lfloor \begin{array}{c} \sum_{i=1}^{p}\alpha_{11i}\\ \sum_{i=1}^{p}\beta_{21i}\\ \sum_{i=1}^{p}\gamma_{31i} \end{array}\begin{array}{c} \sum_{i=1}^{q}\alpha_{12i}\\ \sum_{i=1}^{q}\beta_{22}\\ \sum_{i=1}^{q}\gamma_{32i} \end{array}\begin{array}{c} \sum_{i=1}^{r}\alpha_{13i}\\ \sum_{i=1}^{r}\beta_{23i}\\ \sum_{i=1}^{r}\gamma_{33i} \end{array}\right\rfloor \times \left\lfloor \begin{array}{c} IE_{t-i}\\ R_{t-i}^{+}\\ TO_{t-i}^{+} \end{array}\right\rfloor +\left\lfloor \begin{array}{c} v_{1t}^{+}\\ v_{2t}^{+}\\ v_{3t}^{+} \end{array}\right\rfloor$$

(10) $$\left\lfloor \begin{array}{cc} IE_{t} & \\ R_{t}^{-} & \\ TO_{t}^{-} & \end{array}\right\rfloor =\left\lfloor \begin{array}{c} \alpha_{10}\\ \beta_{20}\\ \gamma_{30} \end{array}\right\rfloor +\left\lfloor \begin{array}{c} \sum_{i=1}^{p}\alpha_{11i}\\ \sum_{i=1}^{p}\beta_{21i}\\ \sum_{i=1}^{p}\gamma_{31i} \end{array}\begin{array}{c} \sum_{i=1}^{q}\alpha_{12i}\\ \sum_{i=1}^{q}\beta_{22}\\ \sum_{i=1}^{q}\gamma_{32i} \end{array}\begin{array}{c} \sum_{i=1}^{r}\alpha_{13i}\\ \sum_{i=1}^{r}\beta_{23i}\\ \sum_{i=1}^{r}\gamma_{33i} \end{array}\right\rfloor \times \left\lfloor \begin{array}{c} IE_{t-i}\\ R_{t-i}^{-}\\ TO_{t-i}^{-} \end{array}\right\rfloor +\left\lfloor \begin{array}{c} v_{1t}^{-}\\ v_{2t}^{-}\\ v_{3t}^{-} \end{array}\right\rfloor ,$$

where, IE, $R_{t}^{+},$ $R_{t}^{-},TO_{t}^{+},andTO_{t}^{-}$ are the variables to be tested in the equation; p. q., and r indicate the optimal lag; and the equation residuals are represented by $v_{1t}^{+}$, $v_{2t}^{+},v_{3t}^{+},v_{2t}^{-},v_{2t}^{-},andv_{3t}^{-}$, respectively.

## Empirical Results and Interpretation

Variable order of integration, that is a test of stationarity, was detected by applying widely used conventional unit root tests, namely, the ADP, P-P, and KPSS tests proposed by Dickey and Fuller (1979), Phillips and Perron (1988), and Kwiatkowski et al. (1992), respectively. The results of the unit root tests are exhibited in Table 2. Study findings unveil that all the researched variables integrated at the level I (0) or after the first difference I (1), but most essentially, neither variables exposed for the order of integration after the second difference, which is desirable.

**TABLE 2:** Conventional *unit* root test.

	**With constant**			**With constant and trend**		
	**ADF**	**PP**	**KPSS**	**ADF**	**PP**	**KPSS**
**Panel A: for Bangladesh**						
IE	−3.322***	−4.319***	0.803***	1.127	–0.597	0.201***
R	−2.241**	−4.823***	0.737***	–1.563	−5.361***	0.094
TO	–0.728	–0.682	0.701***	–1.622	−2.536**	0.118*
ΔIE	0.317	−3.086***	0.709***	−4.514***	−3.402***	0.077
ΔR	−9.739***	−9.24***	0.427***	−9.887***	−9.623***	0.135*
ΔTO	−3.084***	−7.113***	0.123*	−2.407**	−7.018***	0.101*
**Panel B: for India**						
IE	–2.176	−4.52***	0.812***	1.307	–1.204	0.21***
R	–1.514	–1.767	0.723***	–2.337	–2.161	0.096
TO	–0.665	–0.736	0.756***	–2.061	–1.652	0.102***
ΔIE	–0.695	–2.112	0.691***	−2.649***	−2.711***	0.068
ΔR	−8.148***	−7.966***	0.153*	−3.071***	−8.03***	0.074
ΔTO	−5.24***	−5.291***	0.135*	−5.194***	−5.247***	0.134***
**Panel C: for Pakistan**						
IE	–1.071	−7.871***	0.809***	−3.446***	−2.856***	0.212***
R	−2.504***	–1.77	0.181**	–1.937	–1.793	0.166***
TO	–2.309	–2.309	0.298***	−2.731***	−2.608***	0.158***
ΔIE	−3.525***	–1.125	0.727***	−2.522***	–2.151	0.13***
ΔR	–1.991	−5.949***	0.14*	–2.02	−5.989***	0.144***
ΔTO	−6.955***	−7.015***	0.203**	−7.051***	−7.85***	0.165***
**Panel D: for Sri Lanka**						
IE	0.421	–0.544	0.764***	−3.654***	–1.707	0.123***
R	−7.062***	−8.011***	0.667***	−5.736***	−6.233***	0.149***
TO	–1.107	–1.387	0.333***	–1.97	–2.139	0.155***
ΔIE	−3.812***	–2.12	0.783***	−3.806***	–2.106	0.084
ΔR	−4.227***	−4.251***	0.394***	−2.728***	−5.122***	0.131***
ΔTO	−5.194***	−5.194***	0.585***	−4.456***	−5.195***	0.068

The superscript ***, **, and * indicate the level of significance at a 1, 5, and 10%, respectively.

The non-linear unit root test result with Kapetanios et al. (2003) is exhibited in Table 3. The tests were conducted using the raw data (Case 1), the demeaned information (Case 2), and the detrended data (Case 3) for the series. Study findings unveil the research variables: income inequality, remittance, and trade openness, followed by the non-linear process of becoming stationary regardless of the assumption incorporated in the estimation.

**TABLE 3:** Results of KSS non-linear unit root test.

**Series**		**IE**	**R**	**TO**
Case-1	Bangladesh	−4.751***	–0.718	–2.157
	India	−2.751***	−3.124**	0.126
	Pakistan	−6.277***	−3.112**	−6.726***
	Sri Lanka	−6.522***	3.246	−2.898*
Case-2	Bangladesh	−2.517***	−6.774***	−9.654**
	India	−2.728***	−3.373***	−7.528***
	Pakistan	6.142	6.849	−11.672***
	Sri Lanka	6.142	6.214	–2.638
Case-3	Bangladesh	−4.517***	−6.782***	−9.124***
	India	−2.013*	−3.171**	−9.210**
	Pakistan	4.032	7.363***	−10.890***
	Sri Lanka	4.032	7.634***	−6.811***

	**level**	**Case-1**	**Case-2**	**Case-3**

**Critical value** Kapetanios et al. (2003)				
	1%	−2:82	−3:48	−3:93
	5%	−2:22	−2:93	−3:40
	10%	−1:92	−2:66	−3:13

The superscript ***, **, and * indicate the level of significant at a 1, 5, and 10%, respectively.

More so, before our discussions in section “Data and Econometric Methodology,” we did mention that Kapetanios et al. (2003) assumed the test location parameter “c” to be zero (0). At the same time, Kruse (2011) shows that the possibility of a non-zero location parameter is imminent in real-world examples. Hence, he extends the test to allow for a non-zero location parameter. However, as in Kapetanios et al. (2003), the tests were conducted using the raw data, the demeaned information, and the detrended data for the series under investigation.

The results of the Kruse (2011) non-linear unit root test are displayed in Table 4. The linear unit root test’s null hypothesis is rejected at either a 1 or 5% significance level, implying that the series of income inequality, remittance, and trade openness follow non-linear stationary processes.

**TABLE 4:** Results of Kruse non-linear unit root test.

**Series**		**IE**	**R**	**TO**
Case-1	Bangladesh	24.943***	0.921	1.634
	India	35.526***	8.064	10.929*
	Pakistan	12.841***	4.575	15.115**
	Sri Lanka	9.874**	38.126***	5.664
Case-2	Bangladesh	14.009***	13.064***	17.198***
	India	11.267***	16.524***	9.383
	Pakistan	5.947	3.280	13.954**
	Sri Lanka	15.748***	13.046***	6.286
Case-3	Bangladesh	16.952***	12.243***	16.048**
	India	30.948***	5.748	7.150
	Pakistan	11.287***	3.780	3.101
	Sri Lanka	14.214***	11.332***	5.807

		**Case-1**	**Case-2**	**Case-3**

**Asymptotic critical values of t-statistic**				
	1%	13.15	13.75	17.10
	5%	9.53	10.17	12.82
	10%	7.85	8.60	11.10

Notes: The critical values are from Kruse (2011). A denotes the optimal lag length selected by the SBC. The estimation and tests were conducted using a program code written in “R” produced by Kruse. ***, **, and * denote rejecting a unit root’s null at the 1, 5, and 10% significance level, respectively. Non-linearity test.

The following two estimations deal with the investigation of the presence of non-linearity in the empirical model. First, the null hypothesis, irrespective of dimension, is rejected at a 1% significance level. See panel A of Table 4. Second, this suggests a non-linear relationship between remittance, trade openness, and inequality conclusion for all sample countries.

Furthermore, the assessment of non-linearity is also investigated through the application of non-linear OLS. Panel B of Table 5 exhibits the results of non-linear OLS. The null hypothesis of linearity in the empirical model was rejected at a 1% significance level, implying that the relationship between remittance, trade openness, and inequality follows a linear trend.

**TABLE 5:** Results of Brock–Dechert–Scheinkma (BDS) and NOLS.


	**Bangladesh**			**India**			**Pakistan**			**Sri Lanka**		
**Dimension**	**BDS Stat**	**Std. Error**	**z-Stat**	**BDS Stat**	**Std. Error**	**z-Stat**	**BDS Stat**	**Std. Error**	**z-Stat**	**BDS Stat**	**Std. Error**	**z-Stat**
**Panel A: BDS statistics for non-linearity**												
2	0.080***	0.007	10.218	0.003	0.010	0.351	0.018	0.009	1.958	0.043	0.009	4.377
3	0.141	0.012	11.169	0.017	0.017	1.028	0.040	0.015	2.613	0.052	0.015	3.315
4	0.188	0.015	12.394	0.010	0.021	0.491	0.039	0.018	2.095	0.056	0.019	2.887
5	0.212	0.016	13.223	0.028	0.022	1.264	0.040	0.019	2.053	0.049	0.020	2.382
6	0.217	0.015	13.842	0.029	0.022	1.331	0.036	0.019	1.859	0.041	0.020	2.016

		**Bangladesh**			**India**			**Pakistan**			**Sri Lanka**	
		**Variable**	**Coeff**		**t-Stat**	**Coeff**		**t-Stat**	**Coeff**		**t-Stat**	**Coeff**	**t-Stat**

		**Panel B: Non-linear OLS test**												
		R	0.147***		3.159	−0.028***		–0.104	−0.074**		–0.201	0.147**	3.159
		TO	−0.021**		–0.398	0.080***		1.562	−0.274**		–2.745	−0.021***	–0.398
		R^∧^2	0.0173**		0.666	0.031***		1.255	0.068**		0.198	0.017***	0.666
		R^∧^3	−0.076**		–2.180	−0.012***		–0.042	−0.037**		–0.786	−0.076**	–2.180
		R^∧^4	0.032***		0.635	−0.073***		–0.244	0.063**		0.130	0.032***	0.635
		TO^∧^2	0.037***		0.631	0.053***		0.956	0.011***		0.097	0.032**	0.631
		TO^∧^3	−0.029***		–0.388	0.011**		0.153	0.030***		0.230	−0.029***	–0.388
		TO^∧^4	−0.067**		–0.848	−0.019***		–0.242	−0.063***		–0.051	−0.067***	–0.848
		C	4.4063***		40.866	3.697***		9.250	5.014***		10.202	4.406**	40.86
		R-squared	0.936			0.928			0.746			0.794	
		Adjusted R-sq	0.928			0.909			0.722			0.739	
		Wald test	6.597***			7.759***			7.452***			2.679**	
			5.130***			11.188***			0.032			0.752	

The superscript ***, **, and * denote the level of significant at a 1, 5, and 10%, respectively.

The next estimation involves investigating the long-run association by applying the autoregressive distributed lagged, hereafter ARDL, proposed by Pesaran et al. (2001). The ARDL empirical model’s available form is displayed in Equation (11), and the ARDL exhibits results in Table 6.

**TABLE 6:** ARDL cointegration tests.

	**Bangladesh**	**India**	**Pakistan**	**Sri Lanka**
**Panel A: Bound test**				
F-stat	36.711***	8.917***	19.894***	5.312**
t_*BDM*_	−1.84*	−6.397***	−13.364***	−4.789**
**Panel B: Long-run and short-run coefficients**				
LnR	−0.088***	−0.0391***	−0.023**	−0.048***
lnTO	−0.224***	0.127***	0.039***	−0.253***
Δ*ln*R	−0.029**	0.108***	0.984***	0.212***
Δ*ln*TO	0.058**	0.096**	0.067***	0.117***
ECT (-1)	−0.217**	−0.272***	−0.594***	−0.372***
**Panel C: Residual diagnostic test**				
Auto	0.541	0.394	1.064	0.415
Het	0.551	1.297	0.617	0.667
Normality	0.345	1.587	0.794	0.774
Ramsey RESET test	0.664	0.448	0.881	0.807

The superscript ***, **, and * denote the level of significant at a 1, 5, and 10%, respectively.

**Table:** 

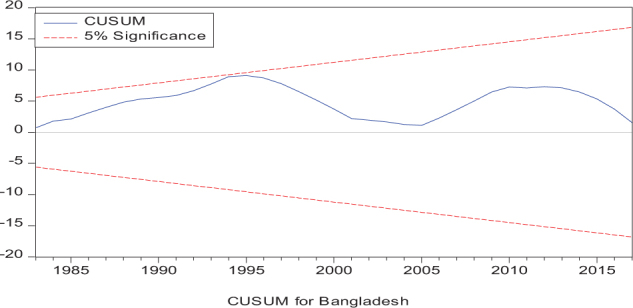
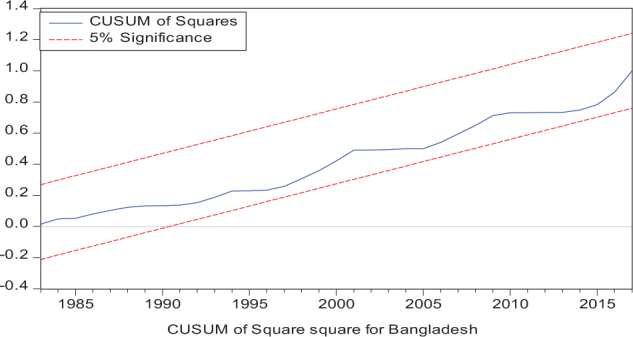
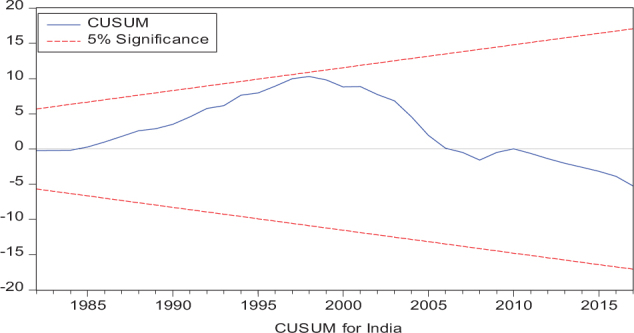
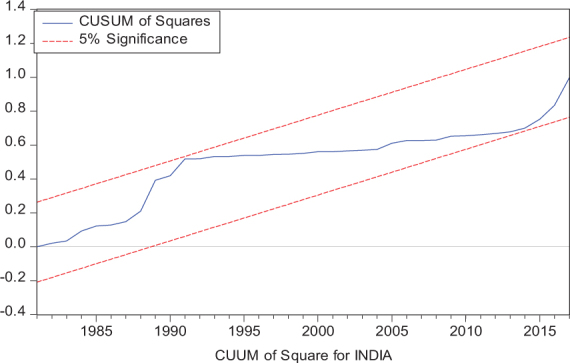
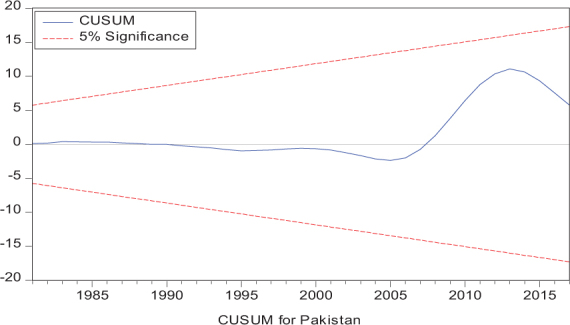
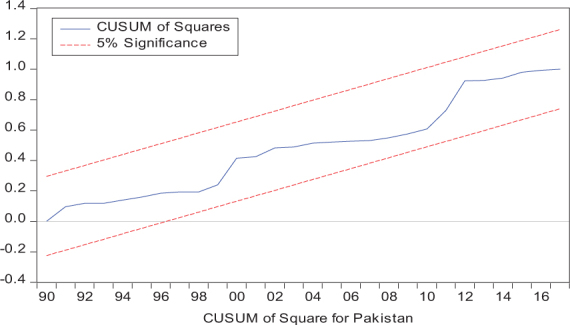
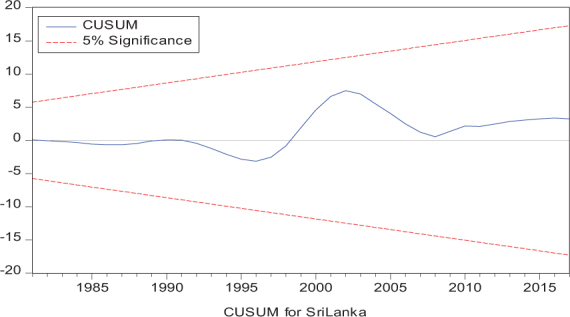
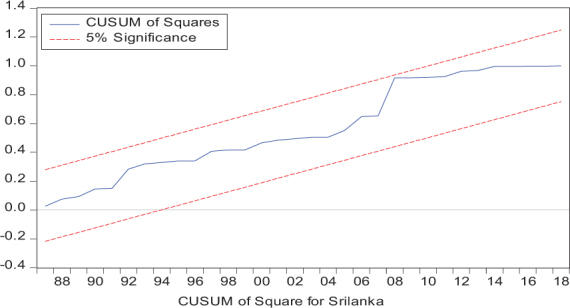
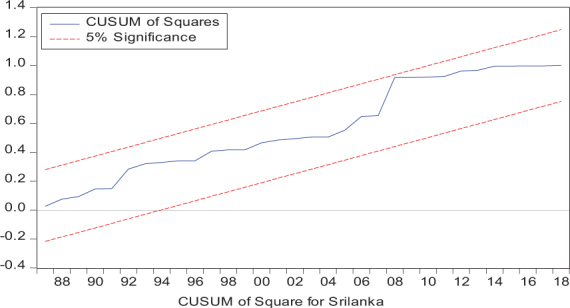
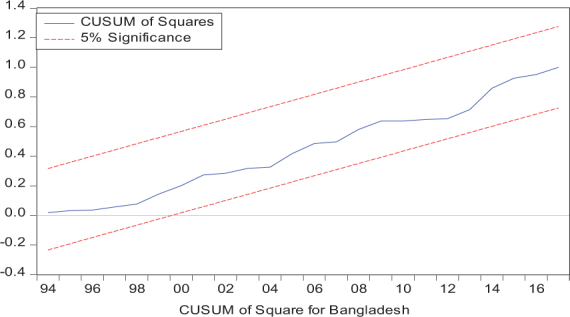
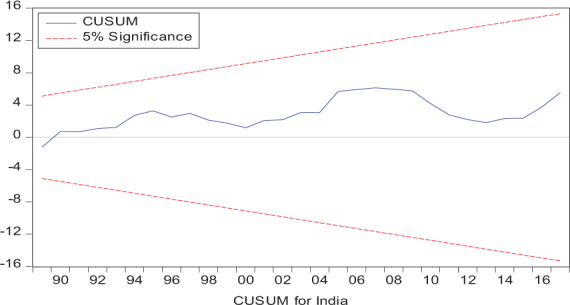
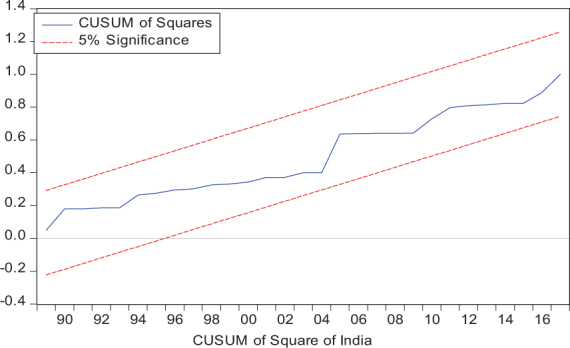
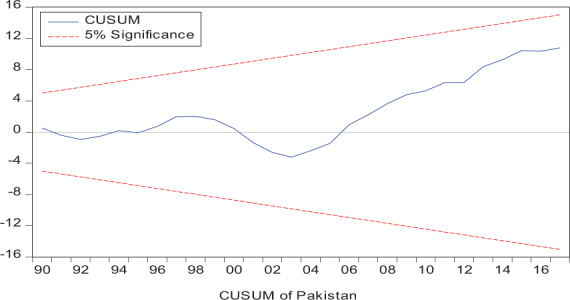
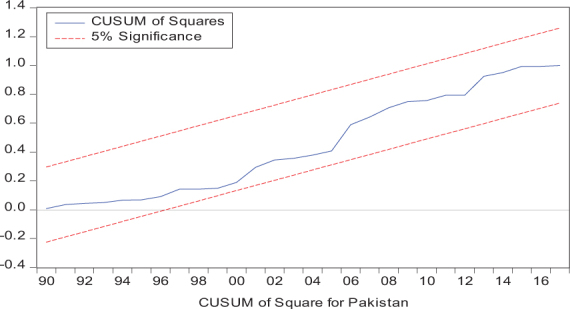
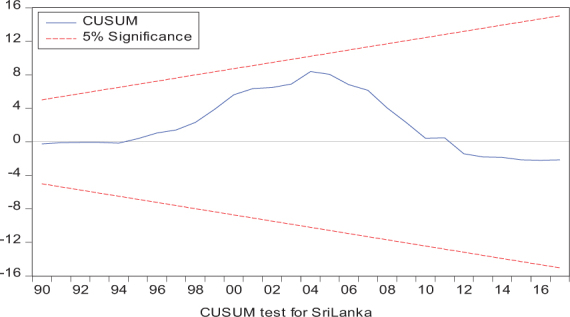
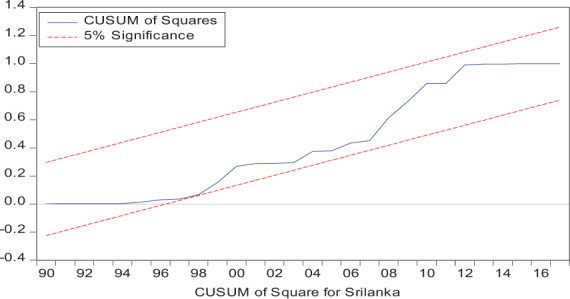


(11) $$△\ln{\left(\mathrm{IE}\right)}_{t}=C_{0}+\theta_{1}△ln{\left(\mathrm{IE}\right)}_{t-1}+\theta_{2}△ln{\left(R\right)}_{t-1}+\lambda_{0}\text{log}{\left(\text{IE}\right)}_{t-1}+\lambda_{1}\text{log}{\left(R\right)}_{t}\lambda_{2}\text{log}{\left(\text{TO}\right)}_{t}+\varepsilon_{t}.$$

Referring to the results of bound testing reported in Panel A, it is evident that there is a long-run relationship between remittance inflows, trade openness, and inequality; this conclusion is valid for each of the sample countries. The long- and short-term magnitudes reported in Panel B, referring to the error correction term’s coefficient, state a long-run association between remittance, trade openness, and inequality. According to long-run magnitude, there is an adverse effect running from remittance inflows to inequality in Bangladesh (a coefficient of −0.488), in India (a coefficient of −0.039), in Pakistan (a coefficient of −0.0233), and Sri Lanka (a coefficient of −0.048), respectively. On the other hand, trade openness exhibits mixed effects running toward inequality, more precisely, the negative effect observed in Bangladesh (a coefficient of −0.224) and Sri Lanka (a coefficient of 0.253) and the positive impact available in India (a coefficient of 0.127) and Pakistan (a coefficient of 0.039).

In the following section, we move to investigate the possible nonlinearity between remittance, trade openness, and income inequality by applying the nonlinear framework proposed by Shin et al. (2014). NARDL, according to Laib and Abadli (2018), Qamruzzaman et al. (2019, 2020), Qamruzzaman and Karim (2020), and Qamruzzaman and Jianguo (2020a), is a new technique that allows modeling asymmetric effects in both the long and the short run by exploiting partial sum decompositions of the explanatory variables (Sadik-Zada et al., 2019; Nguyen et al., 2020). The generalized form of the non-linear empirical model is as follows:


(12) $$△lnIE_{t}=\alpha_{0}+\sum_{i=1}^{n}\mu_{1}△lnIE_{t-i}+\sum_{i=0}^{m}\mu_{2}^{+}△lnPOS{\left(R\right)}_{t-i}+\sum_{i=0}^{k}\mu_{2}^{-}△lnNEG{\left(R\right)}_{t-i}+\sum_{i=0}^{r}\mu_{3}^{+}△lnPOS{\left(TO\right)}_{t-i}+\sum_{i=0}^{j}\mu_{3}^{-}△lnNEG{\left(TO\right)}_{t-i}+\gamma_{0}lnIE_{t-1}+\gamma_{1}^{+}lnPOS{\left(R\right)}_{t-1}+\gamma_{1}^{-}lnNEG{\left(R\right)}_{t-1}+\gamma_{2}^{+}lnPOS{\left(TO\right)}_{t-1}+\gamma_{2}^{-}lnNEG{\left(TO\right)}_{t-1}+\omega_{t}$$

Where, $\left\{ \begin{array}{c} POS{\left(R\right)}_{t}=\sum_{k=1}^{t}lnR_{k}^{+}=\sum_{K=1}^{T}MAX\left(△lnR_{k},0\right)\\ NEG{\left(R\right)}_{t}=\sum_{k=1}^{t}lnR_{k}^{-}=\sum_{K=1}^{T}MIN\left(△lnR_{k},0\right) \end{array}\right.:\left\{ \begin{array}{c} POS{\left(TO\right)}_{t}=\sum_{k=1}^{t}lnTO_{k}^{+}=\sum_{K=1}^{T}MAX\left(△lnTO_{k},0\right)\\ NEG{\left(TO\right)}_{t}=\sum_{k=1}^{t}lnTO_{k}^{-}=\sum_{K=1}^{T}MIN\left(△lnTO_{k},0\right) \end{array}\right.$

The long-run elasticity can figure through, for $R^{+}=\frac{-\gamma_{1}^{+}}{\gamma_{0}}$; $R^{-}=\frac{-\gamma_{1}^{-}}{\gamma_{0}}$; $TO^{+}=\frac{-\gamma_{2}^{+}}{\gamma_{0}}$; $TO^{-}=\frac{-\gamma_{2}^{-}}{\gamma_{0}}$. Similar to linear ARDL bound testing procedure—by F-pass and W-pass statistics under the joint null hypothesis of no cointegration, that is $H_{0}:\gamma_{0}=\gamma_{1}^{+}=\gamma_{1}^{-}=\gamma_{2}^{+}=\gamma_{2}^{-}=0$ and the t*_*BDM*_* statistic, which test the null hypothesis of no cointegration *H*_0_ : *γ*_0_ = 0. When non-linear cointegration is confirmed, the next step is to investigate long-run symmetry $H_{0}=\left(\gamma_{1}^{+}=\gamma_{1}^{-}\right);\left(\gamma_{2}^{+}=\gamma_{2}^{-}\right)$ and short-run symmetry *(additive)* $H_{0}=\left(\sum_{i=0}^{m-1}\mu_{2}^{+}=\sum_{i=0}^{k-1}\mu_{2}^{-}\right);\left(\left(\sum_{i=0}^{r-1}\mu_{3}^{+}=\sum_{i=0}^{j-1}\mu_{3}^{-}\right)\right)$. The results of the NARDL model estimation are exhibited in Table 7.

**TABLE 7:** NARDL cointegration test, long-term, and short-term coefficients.

	**Bangladesh**	**India**	**Pakistan**	**Sri Lanka**
**Panel A**				
F*_*PASS*_*	36.421***	9.793***	33.522***	50.490***
Wpass	13.287***	18.974***	19.889***	35.841***
t_*BDM*_	−16.021***	−7.642***	−37.681***	−6.313***
**Panel B: Long-run and short-run coefficients**				
$R_{LR}^{+}$	−0.129***	−0.126**	−0.119**	−0.152***
$R_{LR}^{-}$	0.018***	−0.052**	0.106***	−0.035***
$TO_{LR}^{+}$	−0.091***	−0.081***	−0.082***	−0.027***
$TO_{LR}^{-}$	0.045**	0.018**	0.144**	0.015**
ECT (-1)	−0.491***	−0.394***	−0.574***	−0.714***
$△R_{SR}^{+}$	0.0793**	0.0488***	0.0118***	0.0949***
$△R_{SR}^{-}$	0.012**	0.0929***	0.0637***	0.06471***
$△TO_{SR}^{+}$	−0.060**	−0.0156**	−0.0483***	0.0494***
$△TO_{SR}^{-}$	0.029***	0.0194**	0.0865***	−0.0285***
*w^R*_*LR*_	9.193***	17.927	3.517***	4.496***
$w_{LR}^{TO}$	6.191***	7.214	12.371***	8.791***
*w^R*_*ER*_	14.512	8.451	8.774	12.411
$w_{SR}^{TO}$	10.541	10.341	9.477	10.274
**Panel C: Residual diagnostic test**				
$X_{auto}^{2}$	0.441	0.794	0.164	0.415
$X_{Heteroskadacity}^{2}$	0.481	0.297	0.517	0.567
$X_{Normality}^{2}$	0.195	0.287	0.694	0.754
Ramsey RESET test	0.564	0.548	0.251	0.473

The superscript ***, **, and * denote the level of significant at a 1, 5, and 10%, respectively.

See Table 7, Panel A. Furthermore, it is revealed that the null hypothesis of long-run symmetry was rejected at a 1% significance level. These findings suggest that the relationship between remittance, trade openness, and inequality follows a non-linear process in the long term.

The results reported in Panel B deal with long-run magnitudes from positive and negative shocks in remittance and trade openness to inequality. Positive shocks in remittance established a negative linkage with inequality, such as a coefficient of −0.129 for Bangladesh, −0.126 for India, −0.119 for Pakistan, and −0.152 in Sri, Lanka, respectively. More specifically, a 10% growth in remittance inflows by migrants in the economy can reduce the present level of inequality in the South Asian economy by 1.29% in Bangladesh, by 1.26% in India, by 1.19% in Pakistan, and by 1.52% in Sri Lanka. Study findings suggest that the future inflows of remittances assist in reducing inequality in the economy. On the other hand, the results of a negative shock in remittances exhibit a positive linkage for Bangladesh (a coefficient of 0.018) and Pakistan (a coefficient of 0.106) and a negative association in India (a coefficient of −0.126) and Sri Lanka (a coefficient of −0.035). In particular, a 10% negative growth in remittances by migrants can augment the state of inequality in sample nations; that is, the level of inequality can be accelerated by 0.18% in Bangladesh, by 1.06% in India, and by 1.26% in Pakistan.

For non-linear effects from trade openness to inequality, the study discloses that positive shocks are negatively associated with Bangladesh (a coefficient of −0.091), India (a coefficient of −0.081), Pakistan (a coefficient of −0.082), and Sri Lanka (a coefficient of −0.027). Findings suggest that the expansion of domestic trade across national boundaries acts as a mitigating factor in reducing the inequality gap in the economy. Furthermore, given a negative shock in trade openness positively associated with inequality, specifically contraction in international business, augments the inequality situation in Bangladesh (a coefficient of 0.045), in India (a coefficient of 0.018), in Pakistan (a coefficient of 0.144), and in Sri Lanka (a coefficient of 0.015), respectively.

The short-run association establishes the error correction term (ECT) coefficient, which is negatively statistically significant, suggesting long-run convergence due to short-run disequilibrium. This refers to the asymmetric effects of remittances on inequality, and study findings document a positive statistically significant linkage between positive shocks in remittances and inequality in Bangladesh (a coefficient of 0.0793), in India (a coefficient of 0.0488), in Pakistan (a coefficient of 0.0118), and in Sri Lanka (a coefficient of 0.0949). Furthermore, the negative shocks in remittances reveal a positive statistically significant linkage with inequality in Bangladesh (a coefficient of 0.012), in India (a coefficient of 0.0929), in Pakistan (a coefficient of 0.0637), and in Sri Lanka (a coefficient of 0.06471). For the asymmetric shocks that are positive and negative innovation in trade openness on inequality, the study establishes positive changes in trade openness negatively linked with inequality in Bangladesh (a coefficient of −0.060), in India (a coefficient of −0.0156), and Pakistan (a coefficient of −0.0483), whereas positive linkage is found in Sri Lanka (a coefficient of 0.0494). Moreover, the negative variations in trade openness disclose a positive statistically significant connection with inequality in Bangladesh (a coefficient of 0.029), in India (a coefficient of 0.0194), and in Pakistan (a coefficient of 0.0865), but a negative connection unveiled in Sri Lanka (a coefficient of −0.0285).

Considering the results of several residual diagnostic tests (see panel C), namely autocorrelation, heteroskedasticity test, normality, and the stability test, they confirm the empirical model estimation reliability and stability, which applies to all four practical models. Furthermore, the CUSUM and CUSUM square test results also produce a similar validity to align with the prior four residual test results. The results of the asymmetry causality test are exhibited in Table 8, in which the impact of independent variables, i.e., positive and negative shocks in remittance inflows and trade openness on inequality.

**TABLE 8:** Hatemi-J asymmetric causality test.

**Null hypothesis**	**Bangladesh**	**India**	**Pakistan**	**Sri Lanka**
*R*^−^≠→*R*^+^	1.916 (0.162)	2.241 (0.121)	4.169**(0.023)	3.535**(0.0390
*R*^+^≠→*R*^−^	3.194**(0.043)	1.325(0.027)**	1.294 (0.286)	2.003 (0.151)
IE ≠→ *R*^+^	9.481***(0.000)	12.74***(0.000)	1.787 (0.182)	9.549***(0.000)
*R*^+^≠→ IE	23.135***(0.000)	3.665**(0.036)	4.588**(0.010)	1.733 (0.191)
IE ≠→*R*^−^	1.840 (0.174)	2.333 (0.112)	2.661*(0.084)	5.756***(0.000)
*R*^−^≠→ IE	8.643***(0.000)	6.226***(0.005)	4.309**(0.021)	11.589***(0.000)
IE ≠→*TO*^+^	2.643*(0.085)	4.213*(0.023)	2.025 (0.147)	0.186 (0.830)
*TO*^+^≠→ IE	6.732**(0.003)	9.156***(0.000)	14.648***(0.000)	8.111***(0.001)
IE ≠→*TO*^−^	5.174**(0.010)	1.562 (0.224)	1.436 (0.251)	5.771***(0.007)
*TO*^−^≠→IE	11.953***(0.000)	2.261 (0.119)	0.131 (0.877)	7.356***(0.002)

The superscripts ***, ** and * denotes the level of significance at 1%, 5% and 10%, respectively.

Considering the results of the causality test, we observe several directional causalities available in an empirical model. However, we concentrate on the critical nexus that we are interested in investigating. First, it is evident that the null hypothesis of positive variation in remittance does not cause inequality is rejected at a 1% level of significance. This finding suggests that additional inward remittance can reduce inequality; this conclusion is valid for all selected countries. Second, the null hypothesis of positive change in trade openness does not because inequality is rejected at a 1% significance level. This finding suggests that trade expansion with internationalization augments consumption and assists in reducing inequality in the economy.

## Discussion

The impact of remittances on income inequality has been extensively investigated in empirical literature by utilizing micro and macro aggregated data and established a mixed order of association. We refer to study findings explaining the nexus between remittances and inequality with both symmetry and asymmetric estimation. It is apparent that continual inflows of remittances positively assist in eradicating the level of inequality in the economy. Study findings align with existing literature, such as Adams et al. (2008b); Anyanwu (2011), and Vacaflores (2018).

Furthermore, prior studies dealing with the South Asian economy support study findings (Uddin and Murshed, 2017; Kumar, 2019). Remittances are anticipated to have a larger impact on lower income nations than developed ones although economic development and inequality may vary (Duval and Wolff, 2016). According to Karpestam (2012), remittances are determined by the recipient nations’ income level, which is either consumption or investment. Furthermore, Pradhan et al. (2008) advocates that remittances boost buying power in underdeveloped nations and support enhancing the standard of living, eventually mitigating the degree of inequality.

Adams and Cuecuecha (2013) demonstrate that international migration and remittances substantially reduce inequality in the developing world, but they do not seem sustainable in the long term. Additionally, they advocate that families receiving remittances spend less on food and more on education, housing, and health, significantly reducing the probability of household inequality. On this premise, remittances improve people’s well-being, mostly via basic spending, but not enough to improve their economic situation. Remittances from immigrants can significantly improve the well-being of the poorest sectors of the population by enabling beneficiaries to raise their consumption, initiate economic ventures, and be more forward-thinking (Vacaflores, 2018). For instance, there is little economic evidence that remittances significantly decrease inequality if a nation does not acquire other financing sources, attract foreign investment, or redirect its absorbing power into economic growth. Remittances may also contribute indirectly to poverty reduction by facilitating access to financial resources for people who would not otherwise be able to engage in the financial system. Giuliano and Ruiz-Arranz (2009) discovered that remittances enable receiving families to fund investment even when they lack access to the official banking system. International remittances have also incentivized receivers to utilize financial instruments (Anzoategui et al., 2011), owing to their lumpy character, which strengthens the financial system and may result in productive investment in the receiving country. Remittances foster financial growth and economic development via increased investment, whether direct or indirect and, therefore, can impact poverty and inequality levels (Akobeng, 2016). The conventional belief is that, in nations with better established financial systems, remittance receivers feel more secure putting their money in financial institutions, allowing them to utilize it for productive projects benefiting a more significant segment of the population (Gupta et al., 2009).

Trade openness and income inequality are currently being investigated, especially in light of new research that disagrees with the widely accepted conventional view that trade expands economic opportunities while simultaneously reducing income disparity (Urata and Narjoko, 2017). Referring to the symmetric and asymmetric effects of trade openness and inequality, study findings establish that domestic trade liberalization assists in lessening income disparity in the economy that is a negative relationship in Bangladesh, which is in line with Daumal (2013) and Aigheyisi (2020). Furthermore, the positive connection is also detected in India, Pakistan, and Sri Lanka by Mahesh (2016) and Chowdhury et al. (2021). Study findings suggest that the impact of trade openness on income inequality immensely relies on the socio-economic condition. Increased openness results in decreased inequality as a result of positive shocks to export demand and trade conditions. It may be a more successful policy approach for reducing inequality in low-income nations (Lim and McNelis, 2016). According to Dollar and Kraay (2003), increasing openness with an improved rule of law results in a larger share of wealth going to the lower classes. Moreover, Ruiz (2017) discovered that some policies help to reduce the gap between the rich and the poor; when nations remove regulatory obstacles to internal competition, free trade, and FDI, this happens.

## Conclusion

In recent decades, the connection between remittances and income disparity has gained keen interest from analysts, economists, and researchers because of the potential of remittances to lead to reducing income inequality. In reality, there is no further disagreement when it comes to the constructive function remittances play in lowering income disparities. In other words, the connection between remittances and income disparity is no longer a disputable problem in finance and economics. The study’s motivation is to investigate the nature of the relationship between remittance inflows, trade openness, and inequality of South Asian countries for 1976–2018. To do so, we performed non-linear tests, including the non-linear unit root test, non-linearity test, non-linear autoregressive distributed lagged (NARDL), and asymmetric causality test. The summary of the key findings of this study are as follows:

First, the non-linear unit root test results following Kapetanios et al. (2003) and Kruse (2011) confirmed that remittance, trade openness, and inequality follow a non-linear process. Furthermore, the non-linearity is investigated through the non-linear OLS and BDS tests proposed by Brock et al. (1987).

Second, the investigation of long-run asymmetry with a non-linear framework is offered by Shin et al. (2014). Study findings from the standard Wald test ascertain that the movement of remittance, trade openness, and inequality is the asymmetry in the long term. Considering the positive and negative shocks in remittance, it is evident that they adversely impact inequality. This finding suggests that excess receipt of remittances decrease inequality through enhancement of money flows in the economy. This effect is available in all sample countries.

Third, directional causality with an asymmetric causality test follows Hatemi-j (2012). Study findings establish bidirectional causality available in Bangladesh for income inequality and positive shocks in remittance inflows [IE←→R^+^;], income inequality and positive shocks in trade openness [IE←→TO^+^], income inequality and negative shocks in trade openness [IE←→TO^–^]. These findings suggest that variability in trade openness in either direction can cause the present state of income inequality. Thus, policymakers should formulate a strategic policy for ensuring continual development in trade internationalization. Moreover, for India, study findings reveal the feedback hypothesis holds for explaining the causality between income inequality and positive shocks in remittances [IE←→R^+^] and income inequality and positive shock in trade openness [IE←→TO^+^] in addition for Pakistan. Study findings disclose a bidirectional association between income inequality and negative shocks in remittances [IE←→R^–^]. Furthermore, in Sri Lanka, bidirectional causality runs between income inequality and negative shocks in remittances [IE←→R^–^] and income inequality and negative shocks in trade openness [IE←→TO^–^]. Furthermore, a number of unidirectional causality is also available, that is, in Bangladesh [R^–^→ IE], in India [R^–^→ IE], in Pakistan [R^+^→ IE; TO^+^→ IE], and in Sri Lanka [IE→R^+^; TO^+^→ IE], respectively.

By taking into account the empirical findings, the study comes up with the following policy suggestions. First, remittance receipts and efficient mobilization have to be confirmed for capitalizing on the benefits to reduce inequality. Efficient reallocation of remittances requires effective financial institutions and efficient intermediation, which support capital accumulation and investment scope in society. Capital accumulation and future investment allow households to increase purchasing capacity and increase their standard of living. Second, the inclusion of remittances recipients into formal financial institutions has to be initiated with adapting innovative financial products and services in the financial system. The inclusion of households in the financial system expands their scope for extra earnings and power to enhance living likelihood. Third, domestic trade liberalization increases the economy’s greater scope of maximizing the scarce economic resources with economic progress; however, international market access increases income disparity with heavy reliance on import concentration. Therefore, trade policies have to be implemented with a focus on lessening income disparity.

Above all, the study finds a non-linear association between remittance inflows, trade openness, and inequality in the selected South Asian countries, namely, Bangladesh, India, Pakistan, and Sri Lanka. Therefore, we conclude that empirical investigation with a non-linear framework might produce more vibrant and robust results and eventually open a new thought avenue for policy formulation by considering a diverse exploration method.

## Data Availability Statement

The original contributions presented in the study are included in the article/Supplementary Material, further inquiries can be directed to the corresponding author/s.

## Author Contributions

LF: introduction, methodology, and first draft preparation. MQ: introduction, methodology, empirical model estimation, and final preparation. Both authors contributed to the article and approved the submitted version.

## Conflict of Interest

The authors declare that the research was conducted in the absence of any commercial or financial relationships that could be construed as a potential conflict of interest.

## Publisher’s Note

All claims expressed in this article are solely those of the authors and do not necessarily represent those of their affiliated organizations, or those of the publisher, the editors and the reviewers. Any product that may be evaluated in this article, or claim that may be made by its manufacturer, is not guaranteed or endorsed by the publisher.
